# A Legacy Question at Cardiff

**Published:** 1914-03-07

**Authors:** 


### A Legacy Question at Cardiff.

### GENERAL LEE'S PROPOSALS.

By reason of a suggestion advanced by Major-General Lee, the chairman of the board of management, exceptional interest was given to the annual meeting of the governors of King Edward VII.'s Hospital, Cardiff. It appeared from the report that, although the income was in excess of that for any preceding year, it was £3,380 less than the expenditure, and that, entirely owing to lack of funds, fifty beds, fully equipped and furnished, remained empty in the new wing of the hospital. General Lee contended that, thanks to the magnificent efforts of Colonel Bruce Vaughan, the investments having now been increased to £129,353, whilst the huge overdraft on the working account had been reduced to £464, a sum of £5,479, awaiting investment, but given or bequeathed for the general purposes of the institution, should be applied to the reduction of the overdraft on the capital or buildings account, which now amounted to £9,147. By that reduction, and bearing in mind their considerable invested capital, he really thought they would be justified in opening the remaining fifty beds, a step which would indeed become necessary when the medical school is fully established. He had recently come across a phrase, "Trustees of posterity," but he thought that *rôle* might be overdone, considering there were never less than 800 sick people appealing at their doors for instant admission; and even regard for posterity should include humane attention to the present generation.

Dr. Herbert Vachell expressed the general regret of the governors at the unavoidable absence of Colonel Bruce Vaughan, but he said Colonel Vaughan had sent him word that he had just received gifts of £500 each from Mrs. John Nixon and Miss Talbot, of Margam, who had already contributed most generously. Dr. Rhys Griffiths, acknowledging a vote of thanks to the honorary medical staff, made a powerful appeal for some wealthy governor or governors to present the hospital with a small quantity of radium, of which there is not a single grain in the institution. He mentioned a recent case where it was desired to relieve a patient's pain, and he was informed that the fee for loan of radium was three guineas per week for ten milligrams. The *Western Mail* has endorsed Dr. Rhys Griffith's appeal, to which wealthy Cardiff should make a quick and generous response. Dr. Ewen Maclean, who followed, stated that it would be a lamentable day if by any great comprehensive stroke of legislation the peculiar characteristics of their voluntary institutions were transmuted into State hospitals.

One of the most appreciated contributions to the meeting was that of Mr. T. J. Hughes, the chairman of the Welsh Insurance Commissioners, who stated they were now within measurable distance of having a great medical school for Wales, which would be second to none in Europe. The medical school would not only be a very great blessing to the Principality but a very good investment for the hospital as well. It was a matter of common experience that where they had medical schools they had brilliant students, full of energy, zeal, and even very helpful to the men who taught them. Cardiff had a unique opportunity. Mr. Hughes paid a high tribute to Sir William James Thomas' liberality and Colonel Bruce Vaughan's unforgettable services, quoting in this connection the concluding sentences of the report: "It will ever be remembered with gratitude that Colonel Bruce Vaughan, convinced of the nobility of his mission, has laboured unremittingly throughout the years to reach the goal of his generous ambition—the alleviation of human suffering and the promotion of the best interests and aims of medical science." One of the most successful of the annual meetings of the hospital concluded with a vote of thanks to the Lord Mayor of Cardiff, Dr. James Robinson, for presiding, whose presence was especially appropriate, as he is one of the most popular medical men practising in Cardiff.

